# A Role for Phospholipase D3 in Myotube Formation

**DOI:** 10.1371/journal.pone.0033341

**Published:** 2012-03-12

**Authors:** Mary Osisami, Wahida Ali, Michael A. Frohman

**Affiliations:** 1 Center for Developmental Genetics, Graduate Program in Genetics, Stony Brook University, Stony Brook, New York, United States of America; 2 Graduate Program in Molecular and Cellular Pharmacology, Stony Brook University, Stony Brook, New York, United States of America; 3 Department of Pharmacological Sciences, Stony Brook University, Stony Brook, New York, United States of America; Universitat de Lleida – IRBLLEIDA, Spain

## Abstract

Phospholipase D3 (PLD3) is a non-classical, poorly characterized member of the PLD superfamily of signaling enzymes. PLD3 is a type II glycoprotein associated with the endoplasmic reticulum, is expressed in a wide range of tissues and cells, and undergoes dramatic upregulation in neurons and muscle cells during differentiation. Using an *in vitro* skeletal muscle differentiation system, we define the ER-tethering mechanism and report that increased PLD3 expression enhances myotube formation, whereas a putatively dominant-negative PLD3 mutant isoform reduces myotube formation. ER stress, which also enhances myotube formation, is shown here to increase PLD3 expression levels. PLD3 protein was observed to localize to a restricted set of subcellular membrane sites in myotubes that may derive from or constitute a subdomain of the endoplasmic reticulum. These findings suggest that PLD3 plays a role in myogenesis during myotube formation, potentially in the events surrounding ER reorganization.

## Introduction

Myogenesis is a highly complex, multi-tiered, cell-cell fusion event. Myoblasts, the immature muscle cells, proliferate until they receive intra- and extra-cellular cues to withdraw from the cell cycle, initiate muscle-specific gene expression and fuse together to form nascent myotubes. These nascent myotubes then continue to increase in size and nuclei number by fusing to more myoblasts until they eventually form mature myofibers [1].

During differentiation, myoblast/myotubes make significant changes to their membrane systems to form muscle-specific organelles. The Golgi apparatus disperses into Golgi fragments that associate with myonuclei for localized export of proteins produced by different nuclei [2], [3], [4]. Smooth endoplasmic reticulum (ER) reorganizes itself to form the sarcoplasmic reticulum (SR), which holds and releases calcium for muscle relaxation and contraction [reviewed in 5]. The plasma membrane, called the sarcolemma, forms specialized invaginations called transverse (T) tubules, an extensive membrane system with lipid and protein compositions distinct from the sarcolemma [6]. The T-tubules function to transfer action potentials to the SR, eventually leading to muscle contraction.

Expression profiling of differentiating C2C12 myoblasts revealed upregulation of Phospholipase D3 (PLD3) during myogenesis [7]. PLD3 is a lesser known family member of the PLD superfamily, a family of phospholipases found in organisms from bacteria to mammals. The extensively studied classical mammalian PLDs, PLD1 [8] and PLD2 [9] play vital roles in vesicle trafficking and fusion events in a number of cellular activities by producing phosphatidic acid from phosphatidylcholine [10], [11], [12], [13], [14], [15]. Other members of the PLD superfamily have been shown to be involved in fusion events. Spo14, the yeast PLD homolog, is required for the fusion of the prospore membrane [16], and mammalian and drosophila mitoPLD participate in mitochondrial fusion and fission [17], [18], [19].

PLD3 has not been well-characterized, in part due to its divergence from the classical PLDs such as mammalian PLD1 [8] and PLD2 [9], which function enzymatically via the action of a pair of HxKxxxxD/E “HKD” split-catalytic domains that define members of the PLD superfamily [20]. PLD3 also has a pair of HKD domains, however neither canonical activity nor substrate have been identified for it [21], [22]. PLD3's closest homolog is the K4L protein found in vaccinia virus. Little can be inferred from the homologous relationship with the vaccinia virus K4L protein because vvK4L is not critical for the viral life cycle and no *in vivo* function has been identified for it [23]. *In vitro* studies have implicated vvK4L in nicking and joining of viral DNA [24], but because this putative nuclease and repair activity is not required for viral growth in tissue culture or in mice, it is presumably redundant in those settings. The closest homolog of PLD3 and vvK4L with a known function is the vaccinia virus F13L protein, which is required for efficient viral cell-cell spreading by wrapping intracellular virions in *trans*-Golgi or endosomal membranes [23], [25], [26]. Like other PLDs, F13L requires an intact HKD domain for activity [25], [26], and the function performed by F13L is blocked by 1-butanol, a crude chemical inhibitor of classic PLD activity, but not by 2-butanol, a control alcohol that does not block classic PLD activity, suggesting that F13L has PLD-like activity. However, overexpression of PLD1 does not rescue F13L-deficient virions [25], suggesting that the role(s) of F13L is more complex than to simply produce PA in the Golgi or other endosomal membranes. F13L has also been suggested to be a broad spectrum lipase with phospholipase A and phospholipase C activities [25].

PLD1 and PLD2 localize to membrane structures via their PX or PH domains [27]. PLD3 does not encode a PX or PH domain, but has been shown to localize to the ER as a transmembrane protein [21], presumably via a predicted transmembrane domain at the N-terminus or a potential prenylation site at the C- terminus [22], although these have not examined experimentally.

Expression studies have revealed that in addition to becoming upregulated during myogenesis, PLD3 also increases in expression in brain and neural tissues during neural differentiation [21], [22]. These findings suggest that PLD3 may be generally involved in cellular differentiation events. PLD3 has also been implicated in sensing oxidative stress [28], which can impact differentiation events such as myogenesis [29]. In the work we describe here, we find that an increase in PLD3 expression facilitates myoblast differentiation, whereas expression of a dominant-negative PLD3 mutant allele delays differentiation. We hypothesize that this may be through the sensing of ER stress since PLD3 expression increases as a consequence of exposure to agents that cause ER stress and are known to promote myogenic differentiation [30]. These are the first functions found for PLD3, and in particular in myoblast differentiation.

## Materials and Methods

### Cell culture and differentiation

C2C12 myoblasts [7] and NIH 3T3 fibroblasts [8] were maintained in a humidified incubator containing 5% CO2 at 37°C and grown in DMEM supplemented with 10% fetal bovine serum (FBS), henceforth known as proliferation media (PM). To induce myogenic differentiation, C2C12 myoblasts were grown, until confluent, in proliferation media. Once confluent, the myoblasts were switched from PM to differentiation media (DM), which was DMEM supplemented with 2% horse serum (HS).

### Plasmid transfection and infection

All DNA transfections, except viral packaging cell transfections, were conducted using Lipofectamine and Plus reagents (Invitrogen) and 1 µg of DNA following the manufacturer's instructions.

For infections, Plat-E [31] cells were transfected using calcium phosphate. 48 hours after transfection, viral supernatants were collected. Cells were infected by centrifuging the cells for 1 h at 1800 RPM in the collected viral supernatant supplemented with 8 µg/mL polybrene. 48 hours after infection, the cells were selected with puromycin.

An open reading frame for human PLD3 was amplified from a cDNA pool, subcloned into pcDNA3, and verified by DNA sequencing. PLD3 was subcloned into additional vectors as described; plasmids described in this report and additional details regarding cloning will be provided upon request.

### Quantitative PCR

C2C12 myoblasts were allowed to differentiate for 6 days as stated above with cell pellets collected on a daily basis for the duration of the experiment. RNA was extracted from differentiating myoblasts using the RNeasy mini kit (Qiagen), following the manufacturers directions. 1 ng of total RNA was used for reverse transcription and amplification using the HotStart Sybr green one-step qRT-PCR mastermix kit (USB) and primers specific for PLD3 (FWD 5′ CTGAGGAACCGGAAGCTGT REV 5′ GGAAAGGGGTGGTCCTGA) and GAPDH (FWD 5′ TGGAGAAACCTG CCAAGTATG; REV 5′ GTTGAAGTCGCAGGAGACAAC).

### Cell lysis and Western Blot analysis

Cells were washed in PBS, lysed in modified RIPA buffer (50 mM Tris-HCl, pH 7.4, 1% Triton X-100, 150 mM NaCl, 1% Na-deoxycholate, 0.1% SDS, 1 mM EDTA, 1 mM EGTA 0.5% CHAPS) supplemented with a protease inhibitor cocktail (Roche), and centrifuged at 3000 rpm for 10 min to remove nuclei and cellular debris. The supernatants were then added to 2× sample buffer containing 8 M urea followed by protein separation on 8–15% SDS PAGE gels and transfer onto nitrocellulose membrane. Membranes were blocked with 1% casein in TBS followed by incubation with the appropriate primary antibodies in 1% casein in TBST. Following the primary antibody incubation, membranes were then washed, three times for 5 min each in TBST followed by a one hour incubation with the appropriate secondary antibodies conjugated with either the Alexa 680 (Invitrogen) or IR Dye 800 (Rockland) fluorophores in 1% casein in TBST for one hour. The membranes were then washed 3 times for 5 min each in TBST followed by a 5 min wash in PBS and scanned using an Odyssey Infrared Scanner (LI-COR) to visualize the protein bands.

### Immunofluorescence microscopy

Cells were seeded onto coverslips with or without collagen. When ready, cells were washed 3 times in PBS and then fixed in 4% formaldehyde for 10 min. The cells were then blocked and permeablized in blocking buffer (5%BSA and 5% normal goat serum diluted in PBS) with 0.1% saponin for 1 hour followed by a one-hour incubation in primary antibodies diluted in blocking buffer. Afterwards, the cells were then washed with PBS 3× for 5 minutes each and incubated for one hour in secondary antibodies and DAPI diluted in blocking buffer. The coverslips were mounted on to slides with Vector Mount and visualized by confocal microscopy using either a Zeiss LSM 510 Meta or a Leica SP-2 microscope.

Anti-human PLD3 antisera was generated in rabbits against the peptide YQELKVPAEEPANELPM (amino acids 7–23) as immunogen and affinity-purified. The antisera detected recombinant PLD3 expressed in HeLa cells both in western blots and in immunofluorescent experiments. However, there also appeared to be some residual non-specific immunoreactivity, so the antisera was used only for western blot analysis. A second antisera was generated against the peptide PRFYDTRYNQETPMEIC (amino acids 265–282) but had inferior sensitivity and specificity. Anti-KDEL antibody and other antisera were from Abcam.

### Transient ER stress

Proliferating myoblasts were grown to confluency in PM and treated with DMSO, Brefeldin-A (2 µM), tunicamycin (2 µg/ml), thapsigargin (1 µM), calcium ionophore A23187 (10 µM), or dithiothreitol (1 mM) for 30 minutes to one hour. The cells were washed twice in warm PBS and immediately induced to differentiate by incubating the cells in DM supplemented with insulin (50 nM), changing the media daily for the duration of the experiment.

Differentiated cells prepared on gelatin-coated coverslips were treated as above or with amphotericin B (25 µg/ml), or wortmanin (10 µM) for 4 hours, followed by fixation and confocal imaging.

### Statistical methods description

Statistical analyses were performed using Student's t-test, one-way ANOVA, and Tukey post-hoc.

## Results

### PLD3 protein expression increases during C2C12 myogenic differentiation

**Figure 1:**
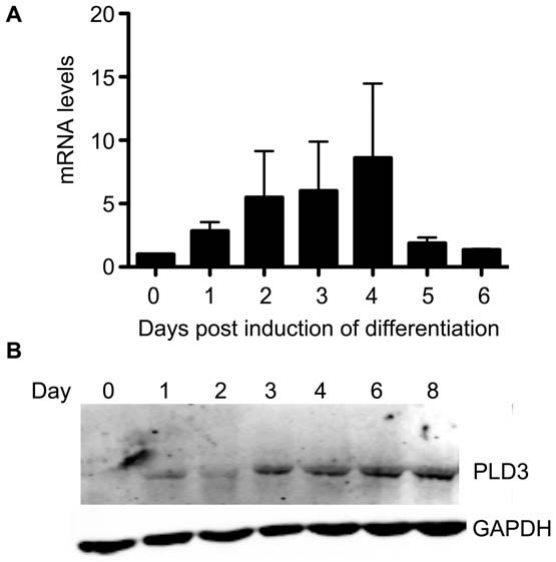
PLD3 expression increases during myogenic differentiation. (**A**) C2C12 cells were induced to differentiate for the indicated periods of time and RNA extracts prepared using RNeasy (Qiagen). Equal amounts of RNA were used as templates in quantitative RT-PCR with PLD3- and GAPDH-specific primers used for amplification, and performed in quadruplicate. The amount of PLD3 mRNA was normalized to the amounts amplified for GAPDH. (**B**) C2C12 cells were induced to differentiate for the indicated number of days and whole cell lysates collected. Equal amounts of lysate protein, as determined by Bradford reagent, were loaded onto SDS-polyacrylamide gels, and the subsequent blot probed with anti-PLD3 and anti-GAPDH antibodies. Representative blot of at least 3 independent experiments.

A prior microarray study had reported that PLD3 mRNA expression increases in C2C12 myoblasts during differentiation [7], which we confirmed in preliminary studies by performing qRT-PCR on RNA collected from differentiating C2C12 myoblasts (Fig. 1A). We then extended this finding by examining whether protein levels similarly increased during differentiation. Whole cell lysates were collected from C2C12 myoblasts and analyzed by western blot analysis, which revealed that PLD3 protein expression levels increased along with mRNA expression during C2C12 myoblast differentiation (Fig. 1B). The PLD3 mRNA levels reached maximal expression at day 4 post-induction of differentiation (Fig. 1A), while the protein levels strongly increased on day 3 and persisted thereafter through day 8 (Fig. 1B).

### The PLD3 transmembrane domain targets PLD3 to the ER

**Figure 2:**
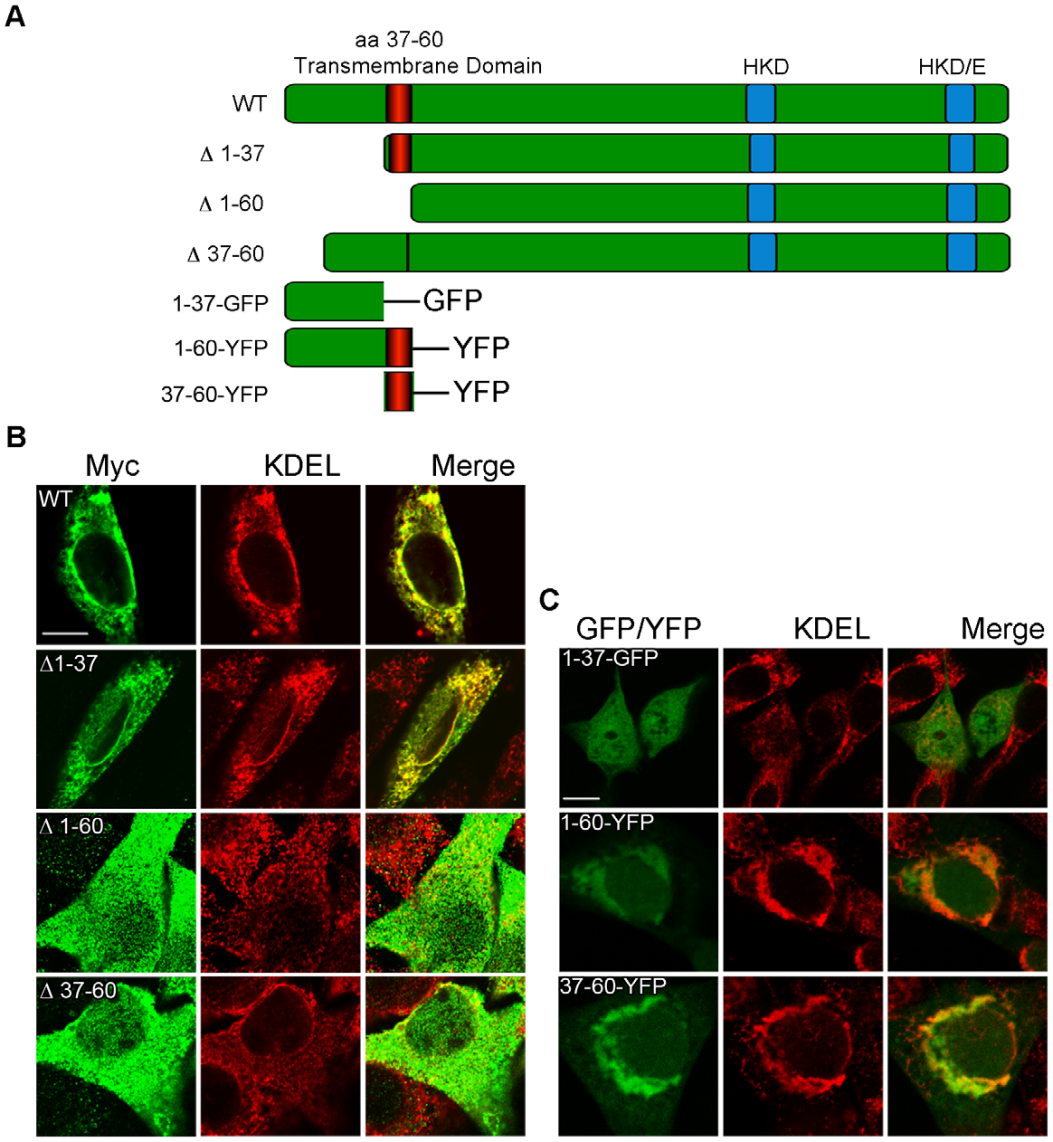
The transmembrane domain directs PLD3 localization in proliferating cells. (**A**) Cartoon schematic of deletion mutants used in this study. (**B**) NIH3T3 cells were transfected with PLD3-myc, PLD3-Δ1–60-myc, PLD3-Δ37–60-myc, or mito-PLD3-Δ1–37-myc and stained with anti-myc and -KDEL antibodies. (**C**) NIH3T3 cells were transfected with PLD3-160-YFP, PLD3-37–60-YFP or PLD3 1–37-GFP and stained with anti-KDEL antibodies. Imaging was performed using a Zeiss LSM 510 confocal microscope. Scale bar, 10 µm. Images are representative of 3 independent experiments.

A prior study had reported localization of PLD3 to the ER and hypothesized that it might be anchored there via a potential N-terminal transmembrane domain starting at amino acid 38 [21]. To test the role of the putative N-terminal signal sequence and transmembrane domain, C-terminally-Myc-tagged mutants were constructed by PCR-based deletion in which either the N-terminal amino acids 1–37(Δ1–37) or 1–60 (Δ1–60) or the transmembrane region amino acids 37–60 (Δ37–60) were removed (Fig. 2A). NIH3T3 cells were transfected with the constructs and immunostained using an anti-Myc antibody to detect the PLD3 mutants and an anti-KDEL antibody to detect the ER. Wild-type (full-length) PLD3 co-localized with the ER (Fig. 2B) consistent with the prior report [21], as did the mutant that retained its transmembrane region, PLD3 Δ1–37-myc, whereas the mutant proteins lacking the transmembrane region (Δ1–60 and Δ37–60) did not, exhibiting instead ubiquitous cytoplasmic localization. These results suggested that the transmembrane region is responsible for PLD3 localization to the ER and constitutes the minimal element required.

To confirm that the transmembrane domain directed PLD3 localization, constructs fusing the relevant N-terminal fragments of PLD3 to EGFP or YFP were constructed and analyzed as above. The fusion proteins containing the transmembrane domain, PLD3 1–60-YFP and 37–60-YFP, localized to the ER (Fig. 2C), where as the construct containing only the N-terminal amino acids prior to the transmembrane domain (PLD3 1–37-GFP) did not, instead showing a diffuse localization throughout the cell. These results confirm that the transmembrane domain targets PLD3 to the ER and anchors it there.

### PLD3 moves from the ER to ER-associated vesicular-like structures or ER subdomains in C2C12 myotubes

**Figure 3:**
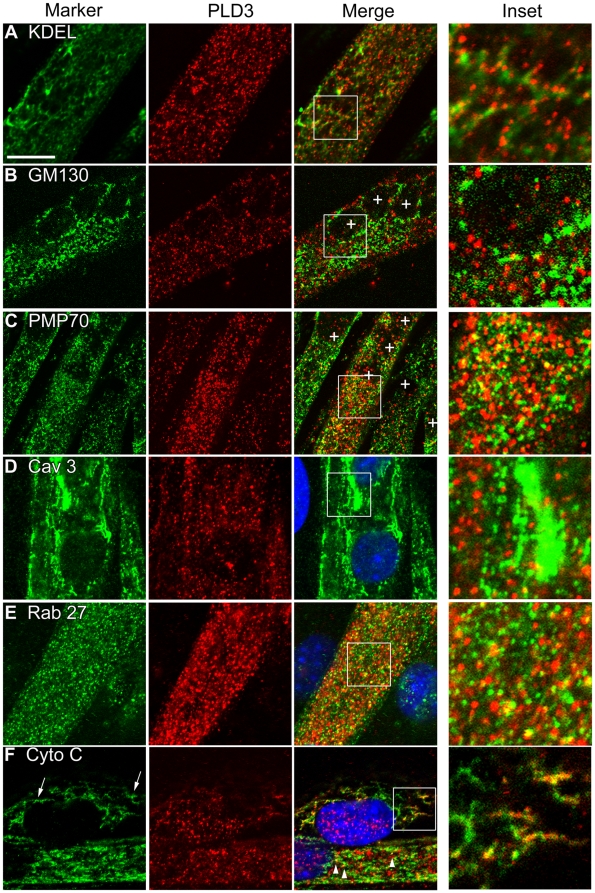
PLD3 localizes to ER-associated vesicles in differentiating myotubes. C2C12 myoblasts expressing PLD3-myc were induced to differentiate for 6 days on gelatin-coated coverslips. Following differentiation the cells were fixed and stained with anti-myc to visualize the recombinant PLD3 protein and (**A**) anti-KDEL, (**B**) anti-GM130, (**C**) anti-PMP70, (**D**) anti-Cav3, (**E**) anti-Rab27 and (**F**) anti-Cytochrome C antibodies. Scale bar, 10 µm. Myoblasts were identified as cells with a single nuclei, as opposed to the multi-nuclei myotubes. Arrows and arrowheads indicate co-localization of PLD3 and mitochondria in myoblasts and myotubes, respectively. Blue, nuclear staining (DAPI); in other panels, asterisks denote nuclei.

Since the localization pattern of a protein in proliferating fibroblasts does not necessarily accurately model its localization in specialized cells, we next set out to examine PLD3 localization in myoblasts differentiated into myotubes. We hypothesized that PLD3 would localize to the ER or an ER-derived membrane system during differentiation since, when overexpressed, it localized to the ER in proliferating NIH3T3 cells (Fig. 2) and in myoblasts (not shown). To determine if overexpressed PLD3 localized to the ER or SR in myotubes, C2C12 cells stably expressing PLD3-myc were differentiated for 6 days. Unexpectedly, minimal co-localization was observed between PLD3 and the ER/SR marker KDEL (Fig. 3A). However, there was a noticeable association between the KDEL-positive ER/SR and PLD3-positive vesicles, in that the great majority of the PLD3-positive vesicles appeared to “decorate” the ER/SR membranes. Potential co-localization was examined for PLD3 and cis-Golgi (using GM130, Fig. 3B), peroxisomes (using 70-kDa peroxisomal membrane protein (PMP70), Fig. 3C), developing T-tubules (using Cav-3 as a marker, Fig. 3D), and secretory vesicles (using Rab 27, Fig. 3E), but no significant association was observed. Other organelle markers such as LC3A/B (autophagosomes) and 488-transferrin (early endosomes) were also scored for co-localization with PLD3, with no overlap observed (data not shown). Examining the relationship between PLD3 and mitochondria was interesting, however. Substantial co-localization was observed in myoblasts undergoing fusion to myotubes between a subset of the PLD3 protein and restricted domains on the mitochondrial tubules (using Cytochrome C to image the mitochondria, Fig. 3F, arrow, inset), resembling the extent of overlap of mitochondrial and ER markers observed at “mitochondrial-associated ER membranes” (MAMs). Co-localization of PLD3 with mitochondria was also observed in the myotubes (arrowheads). These data suggest that PLD3 resides in the ER of non-fused, differentiating/fusing myoblasts before they rearrange their ER membranes, in many cases in close association with mitochondria. Our findings also suggest that PLD3 localizes to ER-derived or highly specialized ER membranes domains lacking KDEL antibody that are in close association with typical ER once fusion takes place. One intriguing possibility involves a recent report that described wrapping of ER tubules around mitochondria tubules to mediate mitochondrial fission [32]; these focal regions are known to be sites of lipid transfer and synthesis, representing potential roles for PLD3.

### Increased PLD3 expression enhances C2C12 myotube formation

**Figure 4:**
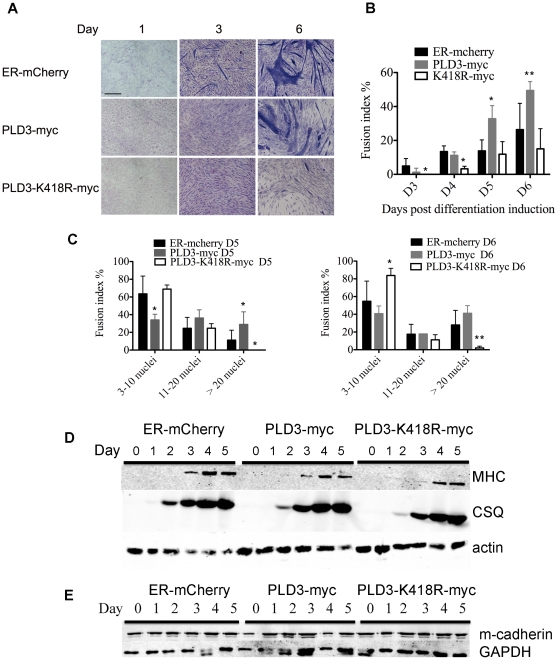
Overexpression of PLD3 promotes myotube formation. (**A**) C2C12 cells expressing PLD3-myc, PLD3-K418R-myc or ER-mCherry were induced to differentiate for 6 days on gelatin-coated coverslips, fixed in methanol, and stained with giemsa. Images are representative photos of 3 independent experiments. Scale bar, 200 µm. (**B**) Total fusion index analysis representing the percent of [nuclei in myotubes with 3 or more nuclei]/[the total number of nuclei] in a field on days (D) 3–6 of differentiation. Ten fields were chosen at random for cell and nuclei counting from 3 independent experiments. *, P<0.05; **, P,0.01. (**C**) Fusion index analysis of myotubes with 3–10, 11–20 or >20 nuclei. (**D, E**) Western blot analysis of MHC, CSQ, and m-cadherin in differentiating C2C12 cells expressing PLD3-myc, PLD3-K418R-myc or ER-mcherry.

**Figure 5:**
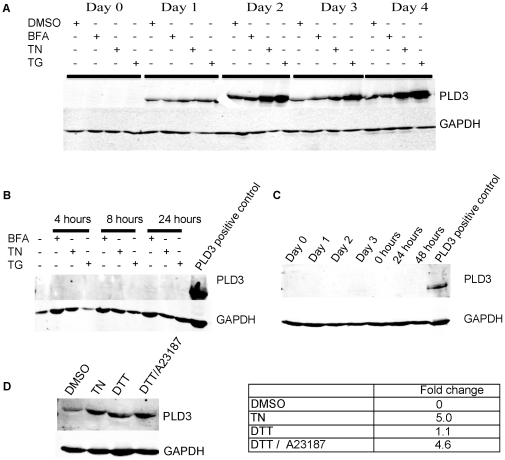
Transient ER-stress increases PLD3 expression during myogenesis. (**A**) Western blot analysis of PLD3 in differentiating C2C12 myoblasts pre-treated for 30 min with thapsigargin (TG, 1 µM), tunicamycin (TN, 2 µg/ml), Brefeldin-A (BFA, 2 µM) or DMSO before differentiation for the indicated periods of time. (**B**) Proliferating C2C12 myoblasts were treated with TG, TN, BFA, or DMSO for the indicated time followed by western blot analysis for PLD3 expression. (**C**) Western blot analysis of HeLa cells pre-treated with TN without (D0) and with (subsequent days) serum reduction for the indicated times. (**D**) Western blot analysis of C2C12 myoblasts pre-treated as shown with DMSO, TN, 1 mM DTT, or DTT and 10 µM calcium ionophore A23187 before differentiation for 3 days. The PLD3 and GAPDH levels of expression were determined using Licor odyssey software. The PLD3 level of expression was normalized to the level of GDPDH expression and is presented as fold-change with respect to the DMSO control. Representatives of three independent experiments are shown.

C2C12 myoblast populations stably expressing PLD3-myc, the presumably catalytically-inactive and dominant-negative PLD3-K418R-myc, or ER-targeted-mCherry (Clontech) as a negative control were generated. Lysine 418 is a key amino acid in the “HKD” catalytic PLD superfamily motif, mutation of which to arginine eliminates enzymatic activity for all PLD isoforms tested thus far [26]. After selection, the cell populations were induced to differentiate for 6 days. Myoblasts expressing ER-mCherry visibly formed myotubes by Day 3 (Fig. 4A) and continued to increase the number and size of the myotubes through Day 6 (Fig. 4B), as scored by a standard version of the fusion index (percentage of [number of nuclei in myotubes with three or more nuclei] over [total nuclei]). Using this fusion index, the PLD3-myc-overexpressing cells exhibited a significant increase in myoblast fusion by day 5–6 compared to the ER-mCherry control cells, while the PLD3-K418R-myc cells exhibited an initial lag in fusion and trended towards lower fusion at day 6. Visually, many of the typical PLD3-myc-overexpressing myotubes also appeared to be larger than the typical ER-mCherry and PLD3-K418R-myc myotubes (Fig. 4A), which was examined quantitatively by determining fusion indices for generation of myotubes with 3–10, 11–20, or >20 nuclei for each of the cell lines. With PLD3 overexpression, there were significantly more myotubes at day 5 with large numbers of nuclei per cell (and fewer with small numbers of nuclei) than in the control myotubes (Fig. 5C), whereas myotubes expressing the presumably inactive PLD3-K418 allele were noteworthy for lacking myotubes with large numbers of nuclei, while having many with only a few nuclei. Taken together, expression of wild-type PLD3 appears to correlate with both an increase in myotube size and an increase in the number of fusion events.

To determine if the change in overall fusion indices correlated with changes in rates of differentiation, temporal expression of the myogenic markers myosin heavy chain (MHC) and calsequestrin (CSQ), were examined by western blot analysis. Control ER-mCherry, PLD3-myc, and PLD3-K418R-myc-expressing myoblasts were induced to differentiate for 6 days and whole cell lysates were collected for western blot analysis. No significant differences in the expression levels of calsequestrin (CSQ) (Fig. 4D) or myogenin (not shown) were observed for the cell lines. There was however, a slight delay in the increase in expression of myosin heavy chain (MHC) in the PLD3-K418R-myc myoblasts. To determine if the change in rates of fusion was due to altered expression of fusion-related proteins, the expression levels of the cell surface myofusion marker m-cadherin were also examined; however, it did not appear significantly different among the three cell populations (Fig. 4E).

### Transient ER-stress with Thapsigargin increases PLD3 expression during differentiation

Transient ER-stress as induced using tunicamycin (TN) or thapsigargin (TG) has been reported to exert a positive effect on myofiber formation increase in culture [30], mimicking the action of signals that drive differentiation in vivo. Given PLD3's pattern of localization in the ER, and the report that PLD3 senses oxidative stress [28] which can impact differentiation events such as myogenesis [29], we examined potential connections of PLD3 to ER stress in myotube formation. Transient ER-stress was induced in C2C12 myoblasts using TN, TG, or Brefeldin-A (BFA) in DMEM supplemented with 20%FBS for 30 minutes to 1 hour immediately before initiating differentiation in DM supplemented with 50 nM insulin. Cell lysates were collected on D0 (day of stress and differentiation induction) and subsequent days for Western blot analysis. PLD3 expression increased by day 2 in the TN- and TG-stimulated myoblasts compared to the non-stimulated control myoblasts, but not in the BFA-stimulated myoblasts (Fig. 5A). The increase in PLD3 expression was sustained throughout the remainder of the culture period, with a maximal PLD3 increase observed of over 8-fold at day 4 for cells pre-treated with TG as determined by densitometry. We next examined if the increase in PLD3 was due simply to ER-stress or the combination of ER-stress and differentiation: ER-stress was induced in C2C12 myoblasts that were exposed to BFA, TN or TG in proliferation medium (PM) for 4, 8 or 24 hours, i.e. without switching to DM to induce differentiation. No induction of PLD3 expression was observed (Fig. 5B), thus, ER stress does not induce PLD3 expression in itself, but rather appears to amplify the induction triggered by differentiation. To confirm the requirement for differentiation, ER-stress was induced in HeLa cells as above that were then cultured for up to 3 days in the reduced serum conditions that stimulate C2C12 differentiation (however, the HeLa cells do not undergo differentiation with this treatment); the HeLa cells were also cultured short-term (up to 48 hours) in PM as above. Neither mode of stress induction led the HeLa cells to upregulate PLD3 expression (Fig. 5C). These observations confirm that the increase in PLD3 expression following ER stress in differentiating myoblasts is due to the combined effects of ER-stress and differentiation.

TN has been show to increase intracellular Ca^2+^ levels and to induce the unfolded protein response (UPR), which in turn causes oxidative stress [33], [34], [35]. To determine if UPR induction and/or increased Ca^2+^ levels stimulate PLD3 expression during differentiation, differentiating myoblasts were pretreated for 30 minutes with the Ca^2+^ ionophore A23187 to stimulate increased calcium levels (data not shown), DTT to activate UPR, or both in combination. The myoblasts were then induced to differentiate for 3 days followed by Western blot analysis to quantitate PLD3 expression. Compared to control myoblasts (Fig. 5D), no significant increase in PLD3 expression was observed for myoblasts pretreated with DTT (1.05-fold change in comparison to control myoblasts, after normalization to the loading control GAPDH) or the Ca^2+^ ionophore A23187 alone (data not shown). However, myoblasts pre-treated with both DTT and A23187 exhibited a 5-fold increase in PLD3 expression that was comparable to that observed for TN, suggesting that it is the combined effect of UPR and increased Ca^2+^ levels that further up-regulate PLD3 expression during differentiation.

**Figure 6:**
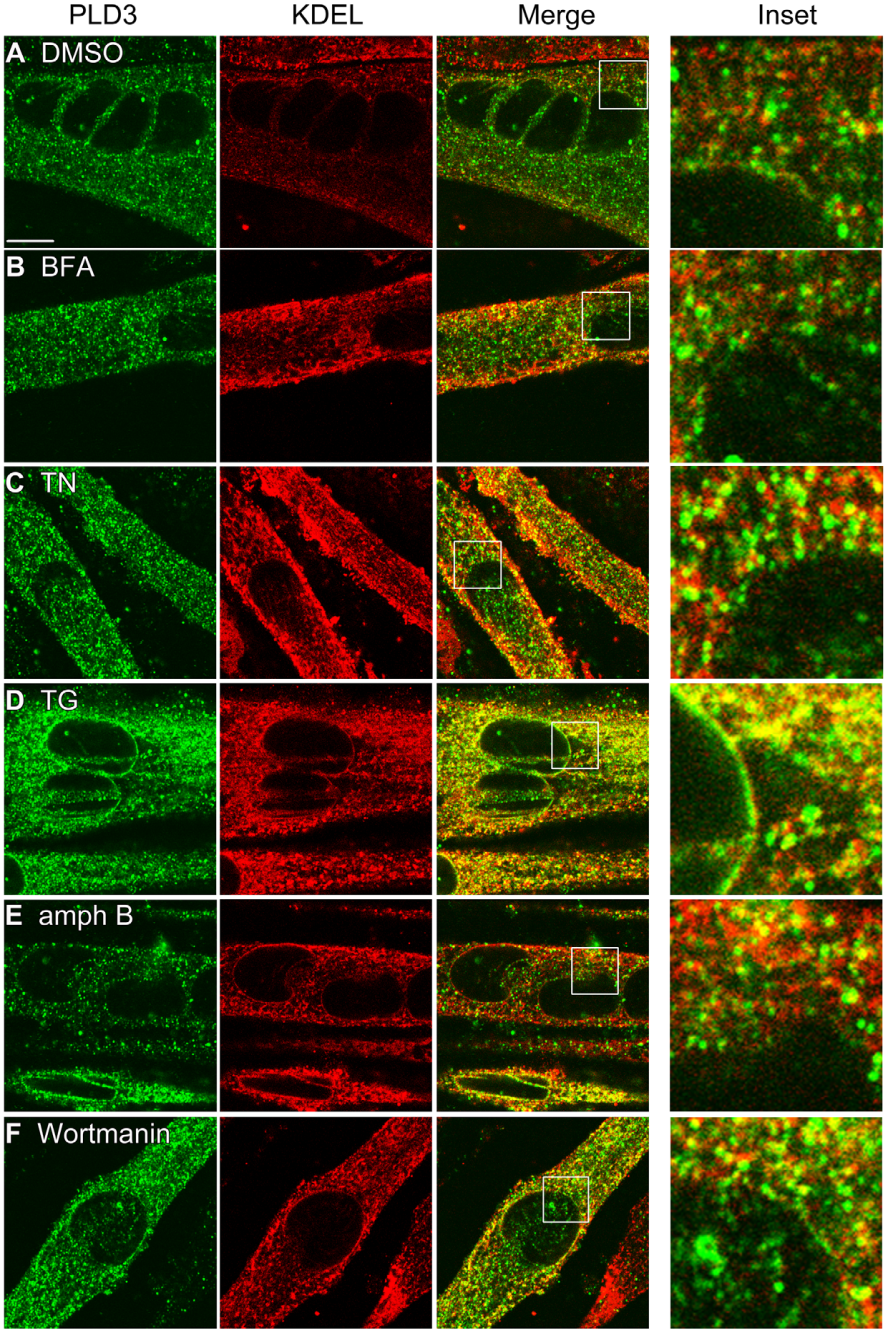
Thapsigargin increases PLD3 localization to perinuclear regions and the ER. C2C12 myoblasts overexpressing PLD3-myc were induced to differentiate for 6 days on gelatin-coated coverslips. After differentiation the cells were treated with (**A**) DMSO, (**B**) BFA (**C**) TN, (**D**) TG, (**E**) amphotericin B (25 µg/ml) or (**F**) wortmanin (10 µM) for 4 hours and then immunostained for anti-myc and anti-KDEL to visualize PLD3 and the ER, respectively.

Since there was an observable change in PLD3 expression in differentiating myoblasts pre-treated with TN and TG, this raised the issue of whether PLD3 might play a role involving altered subcellular localization. To explore this possibility, C2C12 myotubes overexpressing PLD3-myc were allowed to differentiate for 5 days on gelatin-coated coverslips. Once differentiated, the myotubes were treated with BFA, TN, TG, amphotericin B (amph B, which sequesters cholesterol from membranes, leading to the collapse of the developing T-tubule system [36]) or wortmanin (to inhibit PI3-Kinase activity, see [37]) for 4 hours, fixed, stained with anti-myc and -KDEL antibodies, and imaged using confocal microscopy. As initially shown in Fig. 3, PLD3-containing membrane vesicles in control (DMSO-treated) cells were frequently found in proximity to and decorated the ER but did not precisely co-localize with the ER (Fig. 6A). PLD3 localization in myotubes treated with BFA and TN looked largely similar to that seen in the control cells, although a small increase in co-localization was observed (Fig. 6B,C), and myotubes treated with TG, amph B, and wortmanin showed significantly increased amounts of co-localization (Fig. 6D–E). These findings suggest a potential ER-connected role for PLD3 in myotubes for which ER stress has been induced through any of a variety of mechanisms.

## Discussion

Myogenesis is a highly complex, multi-tiered cell-cell fusion event in which myoblasts reorganize much of their cellular membranes and cytoskeleton during the processes that create myotubes and eventually mature muscle fibers. We focused here on PLD3, a poorly understood member of a well-studied family of signaling enzyme, and began by showing that PLD3 protein is upregulated during myoblast differentiation into myotubes, consistent with the increase in PLD3 mRNA noted in a prior microarray study [7]. As previously reported [21] and here in this study, PLD3 overexpressed in proliferating cells localizes to the ER. We have extended this observation to demonstrate that a putative N-terminal transmembrane region is responsible for the subcellular targeting of PLD3, and that a predicted N-terminal secretory sequence is not required. The localization of PLD3 is similar to that recently reported for PLD4 [38], the closest mammalian homolog to PLD3. PLD4 localizes to the ER and Golgi apparatus, presumably also via a transmembrane domain, but it is found in immune cells rather than being expressed in a wide variety of tissues. As with PLD3, no enzymatic activity has been identified for PLD4, and its function is unknown.

Overexpression of PLD3 in differentiating myoblasts increased myotube formation, and conversely, overexpression of a putatively catalytically-inactive allele impeded myotube formation. This is the first reported phenotype for PLD3 and suggests that it may be necessary to look at models for differentiation to explore roles for PLD3 rather than using standard proliferating cell lines. How changes in PLD3 expression/activity alter myotube formation remains unknown; however, the experiments with transient ER-stress induced by TG and TN provide potential insight into the connection.

One basis for how TN and TG affect PLD3 expression might be through control of UPR activation, which in turn has been shown to be involved in several types differentiation events, especially in professional secretory cells [39], [40], [41]. Similarly, TG, which was the strongest inducer of PLD3 expression, has been shown to enhance osteoclast and osteoblast differentiation through activation of oxidative stress response and increase in expression of osteoclast-specific genes [42], [43]. PLD3 and oxidative stress have previously been linked in a report that correlated levels of PLD3 expression to cellular sensitivity to oxidative stress [28].

Numerous attempts to knock down PLD3 and directly examine its role in myogenic fusion were made using siRNA transfection and lentiviral shRNA infection. All of the siRNA approaches were found to be effective in model systems designed for validation of the reagents, e.g. when COS7 cells were transfected with a PLD3 expression plasmid and co-transfected or infected with PLD3 siRNA and lentiviral shRNA, respectively (not shown). RNAi-mediated knockdown of endogenous PLD3 in differentiating myoblasts demonstrated encouraging findings including decreased fusion and cell death. However, PLD3 protein was unexpectedly found to be upregulated in the RNAi-targeted cells. These results are not readily explained by off-target effects given the number of different RNAi targeting sequences employed and the lack of adverse effects in COS7 cells, which lead us to believe that PLD3 is critical for myoblast differentiation. Explanations for these observations remain hypothetical, but one possibility arises from our observation above of a link between ER-stress responses and increased PLD3 expression. It is possible that the RNAi-induced transient decrease in PLD3 protein levels during differentiation led to ER-stress and a strong induction of PLD3 expression/translation and suppression of RNAi machinery, which is an intriguing finding from which to direct future studies.

Taken together, our findings provide evidence to suggest that PLD3 may be important or essential in myotube formation; however, many questions concerning its mechanism of action remain.
